# Heparin-Binding Protein 17/Fibroblast Growth Factor-Binding Protein-1 Knockout Inhibits Proliferation and Induces Differentiation of Squamous Cell Carcinoma Cells

**DOI:** 10.3390/cancers13112684

**Published:** 2021-05-29

**Authors:** Tomoaki Shintani, Mirai Higaki, Tetsuji Okamoto

**Affiliations:** 1 Center of Oral Clinical Examination, Hiroshima University Hospital, Hiroshima 734-8551, Japan; 2 Oral Maxillofacial Surgery, Hiroshima University Hospital, Hiroshima 734-8551, Japan; mirai-higaki@hiroshima-u.ac.jp; 3 Department of Molecular Oral Medicine and Maxillofacial Surgery, Division of Dentistry, Graduate School of Biomedical and Health Sciences, Hiroshima University, Hiroshima 734-8553, Japan; tetsuok@hiroshima-u.ac.jp; 4 School of Medical Sciences, University of East Asia, Shimonoseki 751-8503, Japan

**Keywords:** HBp17/FGFBP-1, FGF-2, squamous cell carcinoma, CRISPR/Cas9, cell differentiation

## Abstract

**Simple Summary:**

Fibroblast growth factor (FGF) plays an important role in tumor growth by inducing angiogenesis in addition to promoting the proliferation of squamous cell carcinoma (SCC) and oral squamous cell carcinoma (OSCC) cells. Heparin-binding protein 17/fibroblast growth factor-binding protein-1 (HBp17/FGFBP-1) purified from A431 cell-conditioned media based on its capacity to bind to FGF-1 and FGF-2 is recognized as a pro-angiogenic molecule as a consequence of its interaction with FGF-2. In this study, we have examined the functional role of HBp17/FGFBP-1 in A431 and HO-1-N-1 cells using the CRISPR/Cas9 technology. Our results showed that *HBp17/FGFBP-1* knockout inhibited cell proliferation, colony formation, and cell motility compared to control. The amount of FGF-2 was decreased in culture medium conditioned by *HBp17/FGFBP-1* knockout cells compared to control. We performed cDNA/protein expression analysis followed by Gene Ontology and protein–protein interaction analysis. The results demonstrate that both gene and protein expression related to epidermal development, cornification, and keratinization were upregulated in *HBp17/FGFBP-1*-knockout A431 and HO-1-N-1 cells.

**Abstract:**

Heparin-binding protein 17/fibroblast growth factor-binding protein-1 (HBp17/FGFBP-1) has been observed to induce the tumorigenic potential of epithelial cells and is highly expressed in oral cancer cell lines and tissues. It is also recognized as a pro-angiogenic molecule because of its interaction with fibroblast growth factor (FGF)-2. In this study, we examined the functional role of *HBp17/FGFBP-1* in A431 and HO-1-N-1 cells. Originally, HBp17/FGFBP-1 was purified from A431 cell-conditioned media based on its capacity to bind to FGF-1 and FGF-2. We isolated and established HBp17/FGFBP-1-knockout (KO)-A431 and KO-HO-1-N-1 cell lines using the clusters of regularly interspaced short palindromic repeats (CRISPR) and CRISPR-associated protein 9 (Cas9) gene editing technology. The amount of FGF-2 secreted into conditioned medium decreased for A431-HBp17-KO and HO-1-N-1-HBp17-KO cells compared to their WT counterparts. Functional assessment showed that *HBp17/FGFBP-1* KO inhibited cell proliferation, colony formation, and cell motility in vitro. It also inhibited tumor growth in vivo compared to controls, which confirmed the significant difference in growth in vitro between HBp17-KO cells and wild-type (WT) cells, indicating that HBp17/FGFBP-1 is a potent therapeutic target in squamous cell carcinomas (SCC) and oral squamous cell carcinomas (OSCC). In addition, complementary DNA/protein expression analysis followed by Gene Ontology and protein–protein interaction (PPI) analysis using the Database for Visualization and Integrated Discovery and Search Tool for the Retrieval of Interacting Genes/Proteins showed that both gene and protein expression related to epidermal development, cornification, and keratinization were upregulated in A431-HBp17-KO and HO-1-N-1-KO cells. This is the first discovery of a novel role of HBp17/FGFBP-1 that regulates SCC and OSCC cell differentiation.

## 1. Introduction

Fibroblast growth factor (FGF) is a cell signaling protein originally isolated from the pituitary gland as a growth factor that promotes fibroblast proliferation [1,2]. So far, 23 members of the FGF family have been identified [3]. Fibroblast growth factor is believed to play an important role in tumor growth and invasion by inducing angiogenesis, in addition to promoting the proliferation of squamous cell carcinoma (SCC) cells [4,5]. The expressions of FGF-1 and FGF-2 increased with the transformation to SCC, and FGF-like activity is also present in SCC cell and salivary gland tumor cell culture mediums. However, since there is no signal peptide in FGFs, its secretory mechanism is unclear [6,7].

Heparin-binding protein 17 (HBp17) was originally co-purified with FGF-2 from culture media conditioned by A431 human epidermoid carcinoma cells, and the molecular weight of HBp17 is 17 kDa [8]. Heparin-binding protein 17, also known as fibroblast growth factor-binding protein-1 (FGFBP-1), binds to FGF-1 and FGF-2 in a noncovalent and reversible manner to induce their release from the extracellular matrix (ECM). The expression of HBp17/FGFBP-1 is observed in epithelial cells, and its expression increased with the severity of epithelial dysplasia [9,10]. In addition, HBp17/FGFBP-1 binds reversibly to FGF-1 and FGF-2 in vitro, so it may be related to the secretion and activation of FGFs and may control cancer cell growth [11]. Liu et al. reported that HBp17/FGFBP-1 complementary DNA (cDNA)-transfected A431-#4, a nontumorigenic A431 clonal variant that does not express HBp17/FGFBP-1, forms palpable tumors in nude mice but parental cells do not [12].

A previous study reported that 1-α,25-dihydroxyvitamin D_3_ (1α,25(OH)_2_D_3_) inhibits HBp17/FGFBP-1 expression in SCC and oral squamous cell carcinoma (OSCC) cell lines via the nuclear factor-kappa B (NF-κB) signaling pathway [13,14]. In addition, ED-71, an analog of 1α,25(OH)_2_D_3_, exhibited antitumor activity against SCC and OSCC both in vivo and in vitro by downregulating HBp17/FGFBP-1 expression [15,16]. The exosomal miR-6887-5p induced by ED-71 inhibits SCC and OSCC cell growth by targeting HBp17/FGFBP-1 [17]. 

In our previous studies, HBp17/FGFBP-1 expression could be reduced but not knocked out completely in cells and in the culture supernatant. In this study, to determine the role of HBp17/FGFBP-1 in SCC and OSCC cells, we investigated the effect of HBp17/FGFBP-1 knockout (KO) using the clusters of regularly interspaced short palindromic repeats (CRISPR) and CRISPR-associated protein 9 (Cas9) gene editing technology on SCC and OSCC cells in vitro and in vivo. For the sake of simplicity, HBp17/FGFBP-1 will be referred to as HBp17 in the remainder of this manuscript.

## 2. Materials and Methods

### 2.1. Cell Culture

We used the epidermoid (squamous cell) carcinoma cell line A431 (RRID:CVCL_0037) and OSCC cell line HO-1-N-1 (RRID:CVCL_1284). Both cell lines were cultured in serum-free medium, as previously described [18,19,20]. Briefly, the cells were routinely grown in 35 mm culture dishes (BD Falcon, San Jose, CA, USA) in DF6F medium composed of a 1:1 mixture, by volume, of Dulbecco’s modified Eagle medium and Ham F-12 medium supplemented with 10 μg/mL of insulin, 5 μg/mL of transferrin, 10 μM of 2-aminoethanol, 10 nM of sodium selenite, 10 μM of 2-mercaptoethanol, and 9.4 μg/mL of oleic acid conjugated with fatty acid-free bovine serum albumin. All chemicals were obtained from Sigma-Aldrich (St. Louis, MO, USA). All reagents used for cell culture were free of mycoplasma and viral pathogens. A431 and HO-1-N-1 cells were cultured at 37 °C in a humidified 95% air/5% CO_2_ atmosphere in a CO_2_ incubator (Thermo Fisher Scientific, Waltham, MA, USA) until they were grown to 60–70% confluency. 

These cell lines are free from mycoplasma contamination and have been authenticated using short tandem repeat profiling (BEX CO., LTD. Tokyo, Japan), and the profiles of A431 and HO-1-N-1 matched with the publicly available reference profiles, as mentioned previously [15,16].

### 2.2. Isolation and Establishment of HBp17-Knockout A431 and HO-1-N-1 Cells

We isolated and established *HBp17*-knockout A431 and HO-1-N-1 cells using HDR-mediated CRISPR KO kits (ORIGENE, Rockville, MD, USA). The reconstructed plasmid, donor template DNA containing homologous arms, and functional cassette were co-transfected into A431 and HO-1-N-1 cells using electroporation. A431 and HO-1-N-1 cells (1 × 10^6^) in 90 μL of DF6F medium were transfected with 5 μg of guide RNA (gRNA) vectors or scramble controls in 5 μL of DF6F medium and 5 μg of the donor DNA in 5 μL of DF6F medium in a 2 mm cuvette using a NEPA21 electroporator (Nepa Gene, Chiba, Japan) at 150 V for 2 min. Two-pulse electroporation was used to transfect exogeneous genes into A431 and HO-1-N-1 cells, and transfected cells were further cultured. Subsequently, 1 μg/mL of puromycin (Life Technologies, Carlsbad, CA, USA) was added to select the cells after subculture of the transfected-A431 and -HO-1-N-1 cells seven times. A single colony was selected, and the inserted DNA sequences were verified by DNA sequence analysis. We designated clones of A431 cells knockout HBp17 as A431-HBp17-KO1 and A431-HBp17-KO2 cells. We also designated clones of HO-1-N-1 cells knockout HBp17 as HO-1-N-1-HBp17-KO1 and HO-1-N-1-HBp17-KO2 cells. Parental cells were designated as A431-wild-type (WT) and HO-1-N-1-WT cells. 

### 2.3. Extraction of RNA and Quantitative Reverse Transcription-Polymerase Chain Reaction Analysis

Total RNA was isolated from A431-HBp17-KO, A431-WT, HO-1-N-1-HBp17-KO, and HO-1-N-1-WT cells using TRIzol reagent (Life Technologies) according to the manufacturer’s instructions, and the RNA quality was checked (RNA concentration > 0.5 µg/µL and OD 260/280 = 1.8–2.0). Reverse transcription was performed using the Super Script first-strand synthesis system (Life Technologies). Quantitative reverse transcription-polymerase chain reaction (qRT-PCR) analysis for HBp17, FGF-2, fatty acid-binding protein 5 (FABP5), small proline-rich protein (SPRR) 1A, SPRR1B, involucrin (IVL), loricrin (LOR), and filaggrin (FLG) was performed using the Stratagene Mx3000P^TM^ system (Agilent Technologies, Santa Clara, CA, USA) with glyceraldehyde-3-phosphate dehydrogenase (GAPDH) as the internal control. Primer sequences and TaqMan^TM^ fluorogenic probes were designed according to ProbeFinder software of the Roche Universal Probe Library system (Roche Applied Science, Nurley, NJ, USA). The primers used are as follows:

- HBp17: (NM_005130) 5′-CGTGTGCTCAGAACAAGGTG-3′, 5′-GAGCAGGGTGAGGCTACAGA-3′ #46 fluorescent probe (Roche Applied Science).
- FGF-2: (NM_002006.4) 5′-TTCTTCCTGCGCATCCAC-3′, 5′-TGCTTGAAGTTGTAGCTTGATGT-3′, #7 fluorescent probe (Roche Applied Science).
- FABP5: (NM_001444.2) 5′-GCAGACCCCTCTCTGCAC-3′, 5′-TCGCAAAGCTATTCCCACTC-3′, #11 fluorescent probe (Roche Applied Science).
- SPRR1A: (NM_001199828.1) 5′-TCGGGTGCATTTGAGGAT-3′, 5′-AAGGAAGACTAGGGATGGTTCA-3′, #60 fluorescent probe (Roche Applied Science).
- SPRR1B: (NM_003125.2) 5′-CAGAGTATTCCTCTCTTCACACCA-3′, 5′-CAAGGCTGTTTCACCTGCT-3′, #3 fluorescent probe (Roche Applied Science).
- IVL: (NM_005547.3) 5′-CCTAGCGGACCCGAAATAA-3′, 5′-GGCCCTCAGATCGTCTCATA-3′, #36 fluorescent probe (Roche Applied Science).
- LOR: (NM_000427.2) 5′-CAGACAAGATGTCTTATCAGAAAAAGC-3′, 5′-GAGGTCTTCACGCAGTCCA-3′, #30 fluorescent probe (Roche Applied Science).
- FLG: (NM_002016.1) 5′-GGACTCTGAGAGGCGATCTG-3′, 5′-TGCTCCCGAGAAGATCCAT-3′, #38 fluorescent probe (Roche Applied Science).
- GAPDH: (NM_020529) 5′-GCTCTCTGCTCCTCCTGTTC-3′, 5′-ACGACCAAATCCGTTGACTC-3′, #60 fluorescent probe.

The expression of each gene was quantified by measuring the cycle threshold (Ct) values and then normalized using the 2^−ΔΔCt^ method relative to GAPDH.

### 2.4. SDS-PAGE and Western Blot

A431-HBp17-KO, A431-WT, HO-1-N-1-HBp17-KO, and HO-1-N-1-WT cells were collected at designated times by scraping them from the surface of the plates in the presence of radioimmunoprecipitation assay (RIPA) buffer (10 mM Tris-HCL, 150 mM of NaCl, 5 mM of ethylenediaminetetraacetic acid (EDTA), 1% Triton X-100, pH 7.4 (Nacalai Tesque Inc., Kyoto, Japan)). The cell suspension was sonicated on ice for 30 s (Taitec Ultra S Homogenizer VP-5S; Taitec, Tokyo, Japan). To prepare the conditioned medium (CM), A431 and HO-1-N-1 cells were cultured in 10 mL of DF6F medium in 100 mm dishes (BD Falcon). The medium was changed to the DF nutrient medium when the cells grew to 80% confluence. Next, the cells were further cultured for 48 h and the CM was collected. The CM was centrifuged at 10,000× *g* for 30 min at 4 °C to remove cells and debris and then concentrated 10 times using an Amicon Ultra-15 Ultracel-5K (Merck). 

Protein content was quantified using a bicinchoninic acid (BCA) assay (Thermo Fisher Scientific). The purified cell extract (5 μg) or the CM (1 μg) was lysed with 5x sodium dodecyl sulfate (SDS) sample buffer (625 mM Tris-HCl (pH 6.8), 10 mM EDTA, 15% glycerol, 0.1% bromophenol blue (BPB)) and electrophoresed under nonreducing conditions on 15% or 11% polyacrylamide gels. The separated proteins were transferred to a polyvinylidene difluoride (PVDF) membrane (Bio-Rad Laboratories, Hercules, CA, USA) using a semidry blotting system (90 mA/gel, Bio-Rad Laboratories). After incubating with 5% skim milk for 1 h at room temperature, anti-HBp17 (Sigma-Aldrich), anti-FGF-2 (Santa Cruz Biotechnology, Santa Cruz, CA, USA), anti-FABP5 (Proteintech Group, Inc., Chicago, IL, USA), anti-SPRR1A (Abcam, Cambridge, UK), anti-SPRR1B (Abcam), anti-IVL (GeneTex, Irvine, CA, USA), or anti-β-actin (Sigma-Aldrich) antibody were used, and the membrane was incubated with peroxidase-labeled secondary antibody (Cell Signaling Technology, Danvers, MA, USA). When analyzing the expression of HBp17 in the CM by Western blotting analysis, 2 ng of recombinant human HBp17 (Santa Cruz Biotechnology) was used as a positive control (PC). The bound antibody was detected with the Clarity Western ECL Substrate (Bio-Rad Laboratories), and chemiluminescence was captured using a ChemiDoc XRS Imaging System (Bio-Rad Laboratories). Original Western blots and densitometric analyses of all blots for quantification of the expression by using the software NIH ImageJ (National Institutes of Health (NIH), Rockville Pike, Bethesda, MD, USA) are shown in Supplementary Figures S3–S5.

### 2.5. Immunofluorescent Analysis

One thousand cells/chamber of A431-HBp17-KO, A431-WT, HO-1-N-1-HBp17-KO, and HO-1-N-1-WT cells cultured in DF6F on the LAB-TEK Chamber Slide (Nulgen Nunc International, Naperville, IL, USA) were fixed with 4% paraformaldehyde for 20 min, washed twice with phosphate-buffered saline (PBS), and blocked with 1% bovine serum albumin (BSA) in PBS for 30 min at room temperature. The cells were stained with anti-IVL at a dilution of 1:100 in 1% BSA in PBS overnight at 4 °C, washed twice with PBS, and then incubated with fluorescent goat anti-rabbit Alexa 488 secondary antibody (Molecular Probes, Inc., Eugene, OR, USA) in 1% BSA in PBS at a dilution of 1:400 for 1 h at room temperature. Next, the cells were covered with VECTASHIELD Antifade Mounting Medium with 4′,6-diamidino-2-phenylindole (DAPI; Vector Laboratories, Inc., Burlingame, CA, USA). Finally, images were taken using the EVOS FL Auto 2 Imaging System (product #AMAFD2000) with an Olympus Super Apochromat objective (×40; product #AMEP4754) and a Nikon Eclipse E800 microscope at ×400 magnification and analyzed using Adobe Photoshop Elements (Adobe Inc., San Jose, CA, USA). The images contained an overlay of IVL (green) and nuclei (blue).

### 2.6. Cell Growth and Colony Formation Assays

We examined the growth of cells in serum-free defined culture. Briefly, A431-HBp17-KO, A431-WT, HO-1-N-1-HBp17-KO, and HO-1-N-1-WT cells were cultured in DF6F serum-free defined medium in 24-well plates (BD Falcon) at a density of 1 × 10^4^ cells/well, and then the cell numbers were counted using a Coulter Counter (Coulter Electronics Inc., Hialeah, FL, USA) daily for 6 days.

For the colony formation assay, A431-HBp17-KO, A431-WT, HO-1-N-1-HBp17-KO, and HO-1-N-1-WT cells were seeded in DF6F medium in 6-well plates (BD Falcon) at a density of 200 cells/well. The cells were cultured for 14 days, and the culture medium was changed every 5 days. The cells were visualized with Giemsa staining solution (Wako Pure Chemical Industries, Ltd., Osaka, Japan), and the number of colonies consisting of more than 50 cells were counted.

### 2.7. Cell Motility Assay

Cell motility was analyzed with a modified Boyden chamber assay using Transwell inserts (6.5 mm diameter) with 8 μm pores (Coaster, Cambridge, MA, USA), as described previously [21,22]. The filters were coated with 100 μg/mL of gelatin (Merck, Darmstadt, Germany) to enhance cell attachment. A431-HBp17-KO, A431-WT, HO-1-N-1-HBp17-KO, and HO-1-N-1-WT cells (5 × 10^5^) were seeded in DF nutrient medium containing 0.1% BSA to the upper compartment of each Transwell insert. After 21 h culture at 37 °C, Transwell inserts were fixed with methanol and stained with Diff Quick (Dade Behringen, Duedingen, Switzerland). The cells on the front surface of the filter were moved with a cotton swab. The cells migrated on the back surface of the filter were stained and cell numbers were counted using light microscopy under a high-power field (×200). The cell numbers in the four fields were counted in each of the three different experiments. Results were expressed as the mean number of migrating cells/mm^2^ ± standard deviation (SD).

### 2.8. Enzyme-Linked Immunosorbent Assay for Soluble FGF-2

We measured the FGF-2 concentration in the 10-fold concentrated CM of A431-HBp17-KO, A431-WT, HO-1-N-1-HBp17-KO, and HO-1-N-1-WT cells. The method of collecting and concentrating the CM was as described above (Section 2.4). The amount of soluble FGF-2 in the CM was measured using a human FGF-2 Quantikine HS ELISA Kit (R&D Systems Inc., Minneapolis, MN, USA) by enzyme-linked immunosorbent assay (ELISA). 

### 2.9. Animal Experiments

We used 4-week-old male athymic Balb/c nude mice (Charles River Japan, Tokyo, Japan). The mice were maintained under specific pathogen-free conditions. All animal procedures were performed in accordance with the Institutional Animal Care and Use Committee guidelines of Hiroshima University (permission #A20-85). All experiments were conducted after the mice were allowed to acclimate to their surroundings for 1 week. One million A431-HBp17-KO and A431-WT cells resuspended in 0.2 mL of DF nutrient medium were inoculated into the flanks of male nude mice, and tumor size was measured twice a week. Tumor volume was calculated by the formula of (1/2 × (major axis) × (the minor axis)^2^). Experiments were performed with five mice.

### 2.10. Microarray Analysis and Gene Ontology Enrichment Analysis

We examined the effect of *HBp17* KO on the cDNA profile of A431-HBp17-KO2 and A431-WT cells. Total RNA was isolated from the cells using TRIzol reagent. The samples were outsourced for cDNA microarray analysis using 3D-Gene (Toray Industries, Inc., Tokyo, Japan). DEGs whose fold changes were <1/2 or >2 in HBp17-KO-2 A431 cells underwent Gene Ontology (GO) functional enrichment analysis (Toray Industries, Inc.). 

### 2.11. Proteomic Analysis

We examined the effect of *HBp17* KO on the protein profile of A431-HBp17-KO2 and A431-WT cells. Cell extracts were isolated with RIPA buffer. The samples were outsourced for proteomic analysis using DIA Proteome Analysis (Kazusa Genome Technologies Inc., Chiba, Japan).

### 2.12. Protein–Protein Interaction Network Construction

We next investigated the functional interactions among DEGs using the online Search Tool for the Retrieval of Interacting Genes/Proteins (STRING, available at https://string-db.org/, accessed on 23 April 2021) database (confidence score > 0.900) [23].

### 2.13. Statistical Analysis

Statistical analysis was performed using BellCurve for Excel (Social Survey Research Information Co., Ltd., Tokyo, Japan). All data were presented as the mean ± SD of at least three independent experiments. Student’s *t*-test was used to compare the difference between two groups. The differences were considered significant at *p* < 0.05.

## 3. Results

### 3.1. Screening and Identification of HBp17 Gene Knockout

**Figure 1:**
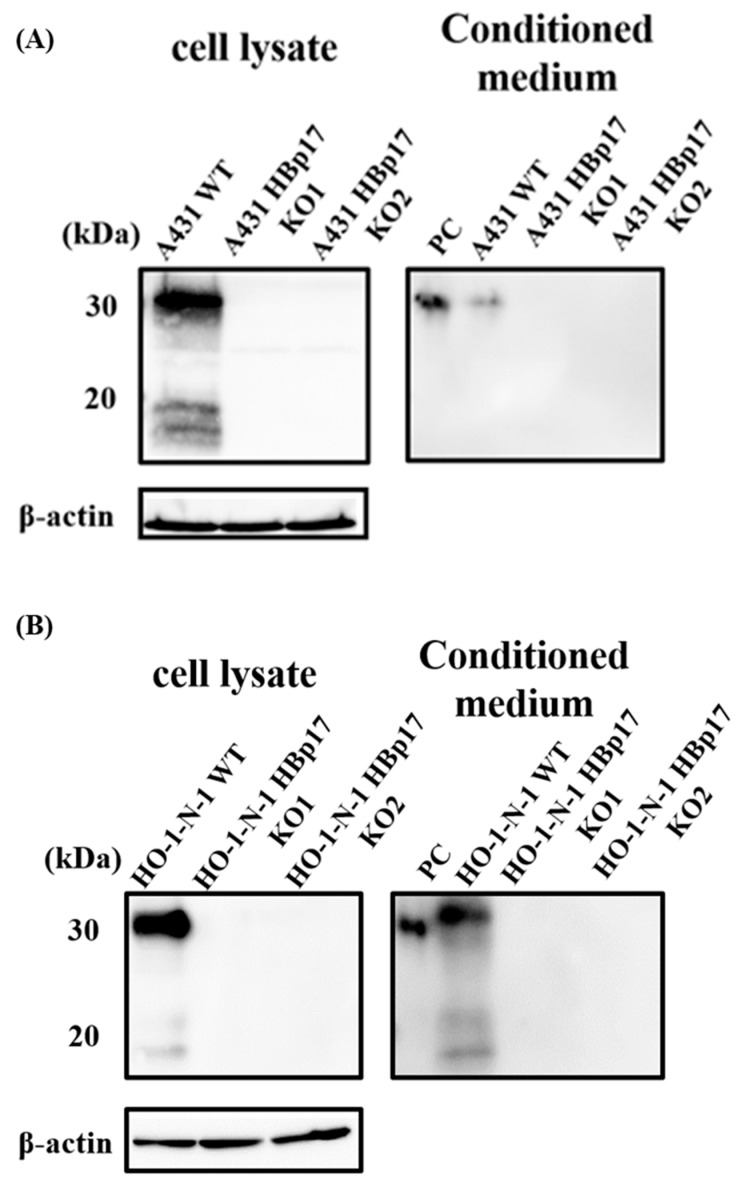
Identification of A431-HBp17-KO and HO-1-N-1-HBp17-KO cells. Cellular protein and CM from A431-HBp17-KO1, -KO2, A431-WT, HO-1-N-1-HBp17-KO1, -KO2, and HO-1-N-1-WT cells were Western blotted against HBp17 goat polyclonal antibody. The absence of the band seen in A431-WT and HO-1-N-1-WT samples confirmed the deletion of HBp17 expression in A431-HBp17-KO (**A**) and HO-1-N-1-HBp17-KO (**B**) cells. Recombinant human HBp17 (2 ng) was used as a positive control (PC).

Two kinds of pCas-Guide-HBp17 guide RNAs (gRNAs) and the functional cassette were co-transfected into A431 and HO-1-N-1 cells by electroporation. The transfected cells were screened by adding puromycin and further cultured. The expression of HBp17 was verified by Western blot. We have successfully knocked out HBp17 at the protein level in cellular protein and in CM of A431 and HO-1-N-1 cells (Figure 1). Clones of A431 cells knockout *HBp17* were designated as A431-HBp17-KO1 and A431-HBp17-KO2 cells. That of HO-1-N-1 cells knockout *HBp17* were designated as HO-1-N-1-HBp17-KO1 and HO-1-N-1-HBp17-KO2 cells. Parental cells used as controls were designated as A431-WT and HO-1-N-1-WT cells. 

**Figure 2:**
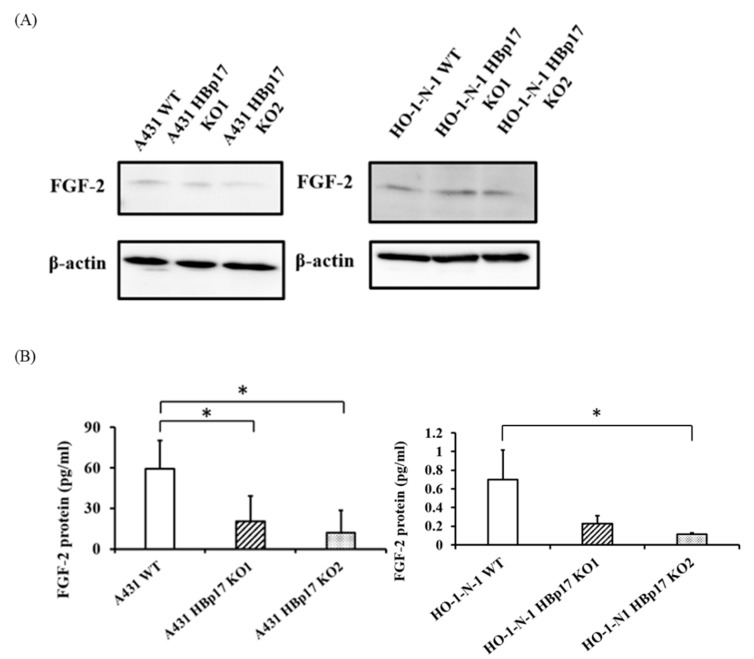
Decreased FGF-2 expression in HBp17-KO cell CM. (**A**) Western blot of FGF-2 in total cellular protein of A431-HBp17-KO1, -KO2, A431-WT, HO-1-N-1-HBp17-KO1, -KO2, and HO-1-N-1-WT cells. (**B**) ELISA of FGF-2 secreted into CM decreased for A431-HBp17-KO and HO-1-N-1-HBp17-KO cells compared to their WT counterparts. Each bar represents the mean + SD. * *p* < 0.05; *n* = 3.

Western blot analysis revealed that there was no difference in cellular FGF-2 levels between HBp17-KO cells (A431-HBp17-KO1, -KO2, HO-1-N-1-HBp17-KO1, -KO2) and A431-WT and HO-1-N-1-WT cells (Figure 2A). However, quantification of FGF-2 in the CM by ELISA revealed that the amount of soluble FGF-2 significantly decreased in HBp17-KO cells compared to the WT cells (Figure 2B). 

### 3.2. HBp17 Knockout Inhibits Proliferation and Colony-Forming Ability of A431 and HO-1-N-1 Cells In Vitro

**Figure 3:**
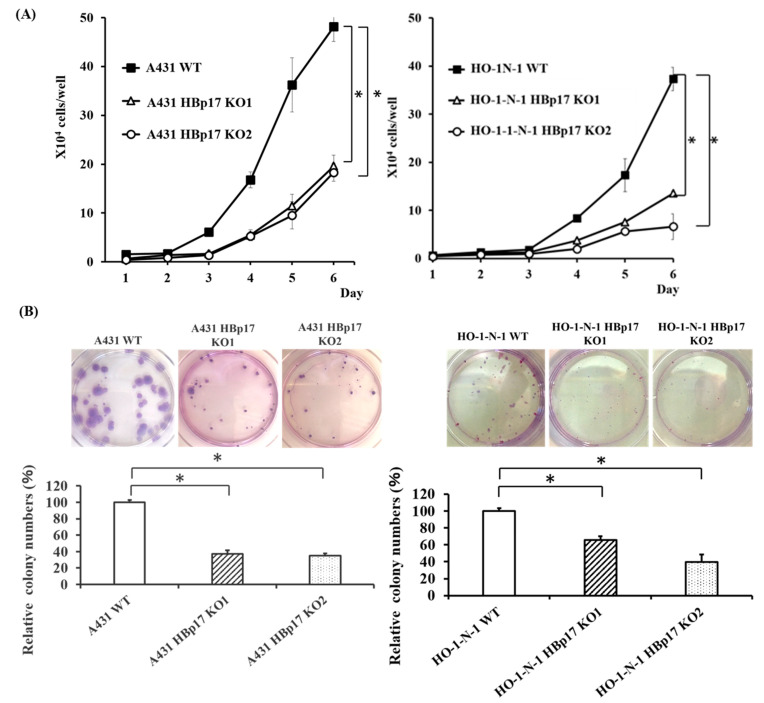
Knockout of HBp17 inhibits cell proliferation and colony formation. (**A**) Cell proliferation and (**B**) colony formation were determined in A431-HBp17-KO1, -KO2, A431-WT, HO-1-N-1-HBp17-KO1, -KO2, and HO-1-N-1-WT cells. Experiments were performed in triplicate with data presented as means ± SD. * *p* < 0.05.

We examined the effect of *HBp17* knockout on A431 and HO-1-N-1 cell proliferation in serum-free defined culture. The proliferation rate of A431-HBp17-KO and HO-1-N-1-HBp17-KO cells was significantly decreased compare to that of A431-WT and HO-1-N-1-WT cells (Figure 3A). 

The effect of *HBp17* knockout on colony-forming ability was also evaluated, and it has been revealed that the ability of A431-HBp17-KO and HO-1-N-1-HBp17-KO cells significantly decreased compared to that of A431-WT and HO-1-N-1-WT cells (Figure 3B).

### 3.3. HBp17 Knockout Inhibits A431 and HO-1-N-1 Cell Motility

**Figure 4:**
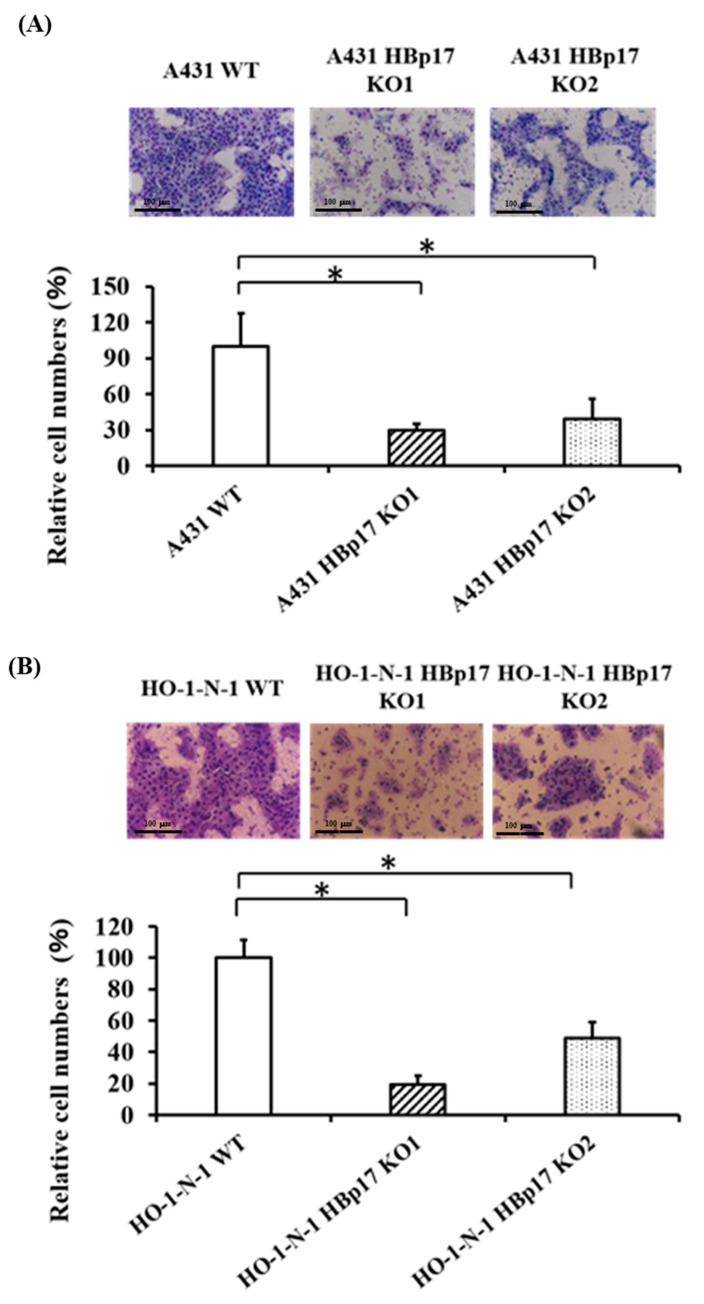
Knockout of HBp17 inhibits mobility of A431 and HO-1-N-1 cells. A431-HBp17-KO1, -KO2, and A431-WT (**A**) and HO-1-N-1-HBp17-KO1, -KO2, and HO-1-N-1-WT (**B**) cells were added to Transwell inserts. Cells migrating across the insert were counted after incubation for 24 h at 37 °C. Experiments were performed in triplicate, with the data presented as means ± SD. * *p* < 0.05.

The motility of HBp17-KO cells and WT cells was evaluated using the modified Boyden chamber method [21]. A431-HBp17-KO cells exhibited significantly decreased cell migration ability compared to A431-WT cells (Figure 4A). Further, motility of HO-1-N-1-HBp17-KO cells also significantly decreased compared to that of HO-1-N-1-WT cells (Figure 4B).

### 3.4. HBp17 Knockout Inhibits Tumor Growth in Athymic Nude Mice

**Figure 5:**
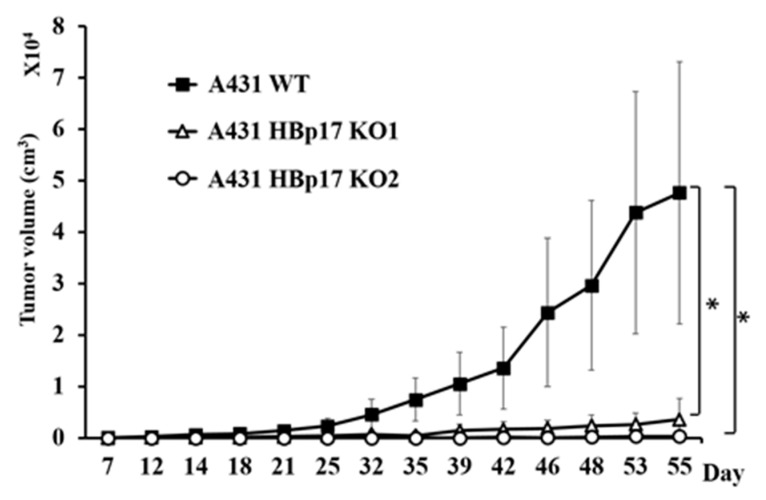
HBp17 knockout suppresses A431 tumor growth in immunodeficient mice. A431-HBp17-KO1, -KO2, and A431-WT cells were inoculated into the flanks of male nude mice, and tumor volume was measured twice a week. Experiments were performed with five mice. Data are presented as mean tumor volume + SD. * *p* < 0.05.

We evaluated the effect of *HBp17* knockout on tumor growth of nude mouse xenografts from the A431-HBp17-KO1, A431-HBp17-KO2, and A431-WT cells. The cells were transplanted into athymic nude mice, and tumor sizes were measured twice a week. The tumor growth of A431-HBp17-KO1 cells clearly decreased compared to that of A431-WT cells (Figure 5). No tumor formation was observed in mice transplanted with A431-HBp17-KO2 cells.

### 3.5. Microarray Analysis and GO Enrichment Analysis

**Figure 6:**
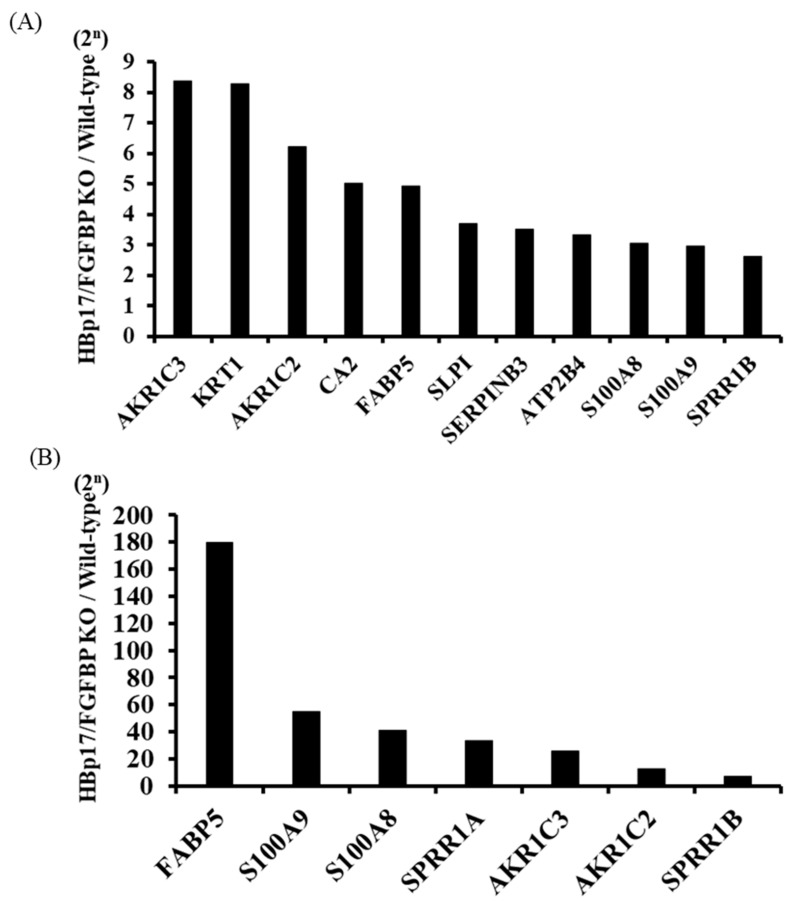
cDNA microarray and proteomic analyses of A431-HBp17-KO2 and A431-WT cells. (**A**) cDNA microarray analysis found 11 cDNAs with greater than 2.5-fold higher expression in A431-HBp17-KO2 cells than in A431-WT cells. (**B**) Seven proteins were found to be expressed at least five times higher in A431-HBp17-KO2 cells than in A431-WT cells. Abbreviations: aldo-keto reductase family 1 member C3 (AKR1C3), keratin1 (KRT1), aldo-keto reductase family 1 member C2 (AKR1C2), carbonic anhydrase 2 (CA2), fatty acid-binding protein 5 (FABP5), secretory leukocyte protease inhibitor (SLPI), serine proteinase inhibitor clade B member 3 (SERPINB3), plasma membrane calcium-transporting ATPase 4 (ATP2B4), S100 calcium-binding protein A8 (S100A8), S100 calcium-binding protein A9 (S100A9), small proline-rich protein 1B (SPRR1B), and small proline-rich protein 1A (SPRR1A).

We examined the effect of knocking out *HBp17* on the cDNA expression profile of A431-HBp17-KO2 and A431-WT cells. We identified 793 Ensemble ID-coded differentially expressed genes (DEGs), of which 347 were upregulated and 446 were downregulated. Statistical analysis comparing cDNA profiles of the cells showed that the expression of 11 cDNAs (aldo-keto reductase family 1 member C3 (AKR1C3), keratin1 (KRT1), aldo-keto reductase family 1 member C2 (AKR1C2), carbonic anhydrase 2 (CA2), fatty acid binding protein 5 (FABP5), secretory leukocyte protease inhibitor (SLPI), serine proteinase inhibitor clade B member 3 (SERPINB3), plasma membrane calcium-transporting ATPase 4 (ATP2B4), S100 calcium-binding protein A8 (S100A8), S100 calcium-binding protein A9 (S100A9), and small proline-rich protein 1A (SPRR1B)) in A431-HBp17-KO2 cells was 2.5–8.4 times higher than that of A431-WT cells (Figure 6A). To clarify the functional and pathway enrichment of DEGs, GO enrichment analysis was applied and the top 10 GO terms of upregulated and downregulated DEGs were selected on the basis of the *p*-value (Table 1 and Table 2). The upregulated genes were associated primarily with cornification, epidermal development, and keratinization, indicating the effects of *HBp17* KO. The downregulated DEGs were associated with cell–cell signaling pathways, such as mitogen-activated protein kinase (MAPK), phosphatidylinositol 3’-kinase (PI3K)/Akt, and extracellular signal-regulated protein kinase (ERK) 1/2.

We focused on the protein–protein interaction (PPI) of upregulated differentially expressed proteins in A431-HBp17-KO2 cells. The analysis of PPI using Search Tool for the Retrieval of Interacting Genes/Proteins (STRING) provided 323 nodes (proteins) and 154 edges (interactions) in the PPI network and identified a main module associated with the formation of a cornified envelope such as Filaggrin (FLG), small SPRR3, Involucrin (IVL), SPRR1A, and SPRR1B (Supplementary Figure S1).

### 3.6. Proteomic Analysis

Proteomic analysis to investigate differential protein expressions identified 5805 proteins, of which 2590 were upregulated and 3215 were downregulated in A431-HBp17-KO2 cells compared to A431-WT cells. The expression of seven proteins such as FABP5, S100 calcium-binding protein A9 (S100A9), S100A8, SPRR1A, aldo-keto reductase family 1 member C3 (AKR1C3), AKR1C2, and SPRR1B in A431-HBp17-KO2 cells was 5 to 180 times higher compared to that of A431-WT cells (Figure 6B). PPI analysis by STRING also provided 157 nodes (proteins) and 93 edges (interactions) in the PPI network and identified 2 main modules associated with the formation of a cornified envelope, including SPRR1A, SPRR2A, SPRR2F, peptidase inhibitor 3 (PI3), SPRR1B, envoplakin (EVPL), SPRR2D, cystatin A (CSTA), plakophilin-1 (PKP1), and keratinization (keratin (KRT) 1, KRT77, KRT7, KRT23, KRT10, KRT85, KRT16, KRT9, and KRT6B) (Supplementary Figure S2).

### 3.7. Validation of Expressions of Terminal Differentiation-Related Molecules in A431-KO and HO-1-N-1-KO Cells

**Figure 7:**
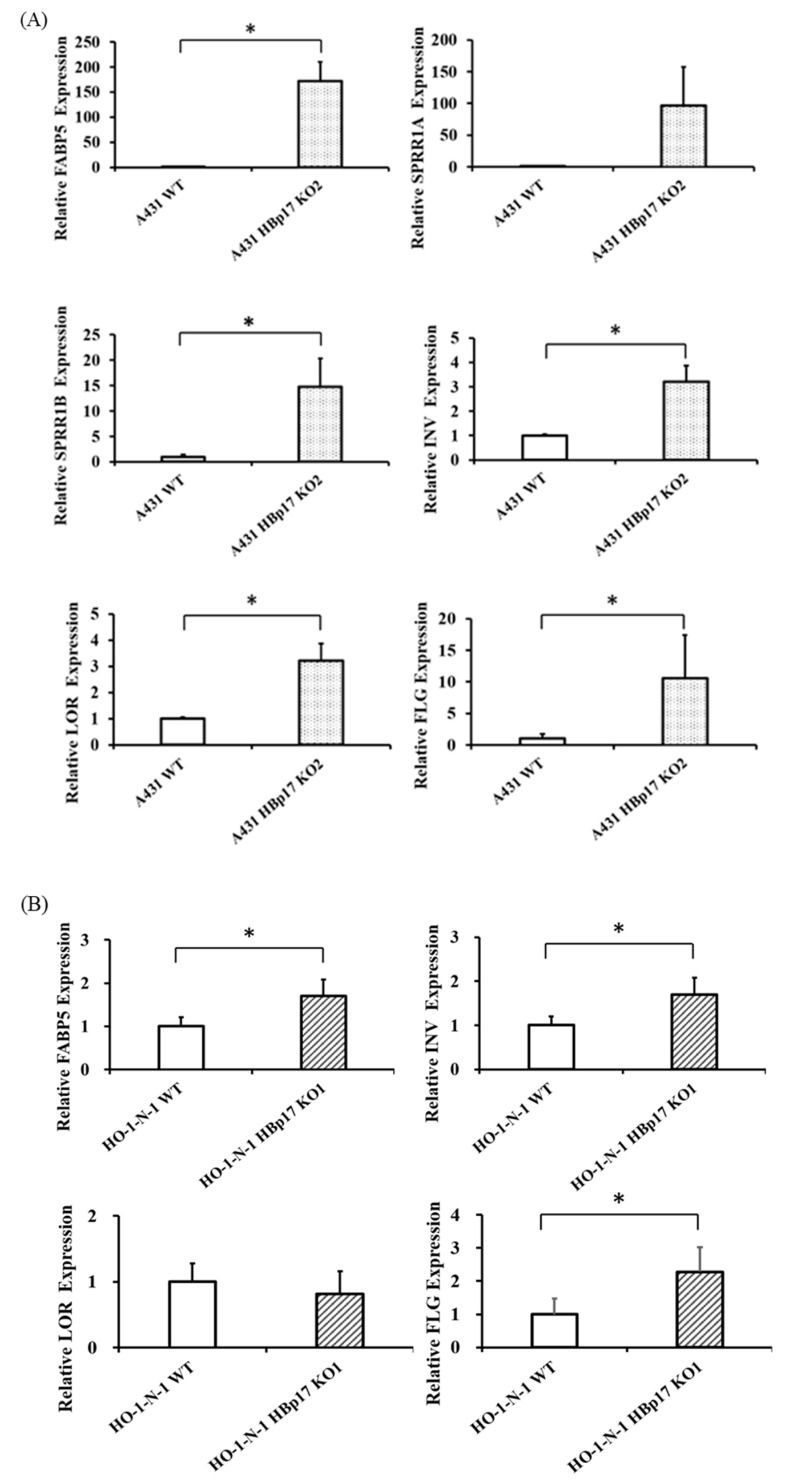

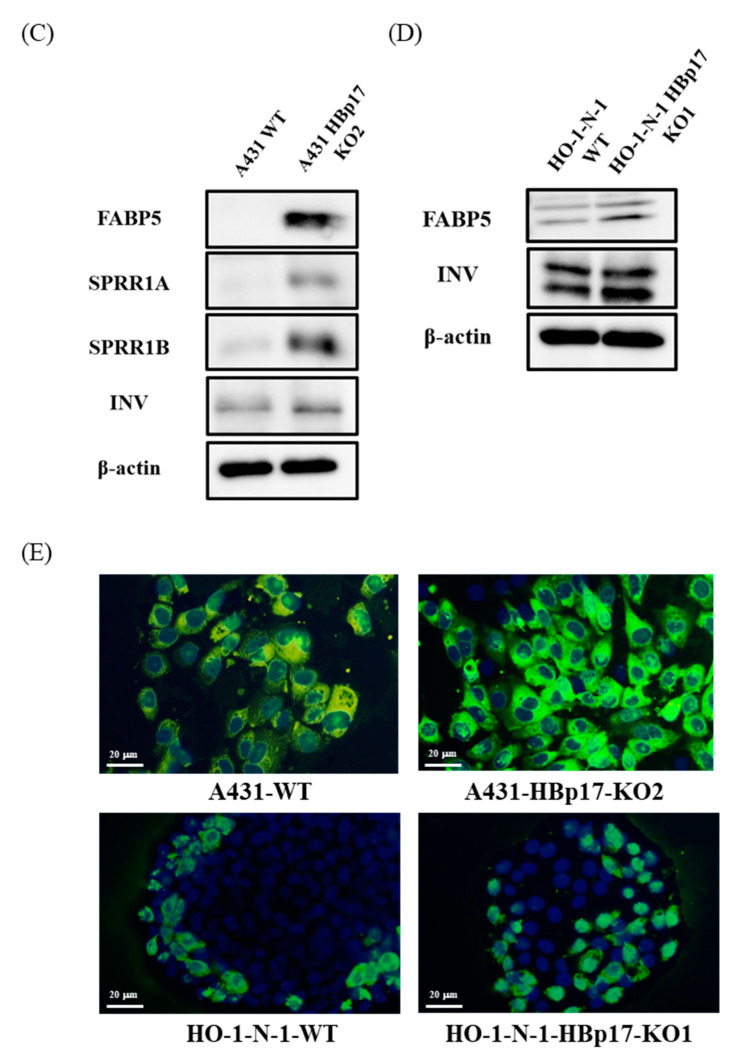
Knocking out HBp17 induces the expression of cornified envelope-related proteins. (**A**) Upregulation of FABP5, SPRR-A, SPRR-B, IVL, LOR, and FLG mRNAs in A431-HBp17-KO2 cells was evaluated by qRT-PCR. (**B**) In HO-1-N-1-HBp17-KO1, the expression of FABP5, IVL, and FLG mRNAs was elevated. GAPDH levels were used as an internal control. Experiments were performed in triplicate, with the data presented as means ± SD. * *p* < 0.05. Increased cornified envelope-related protein expression in A431-HBp17-KO2 (**C**) and HO-1-N-1- HBp17-KO1 cells **(D)** was evaluated by Western blot. (**E**) Immunofluorescence staining for IVL (green) in A431-HBp17-KO2 and HO-1-N-1-HBp17-KO1, A431-WT, and HO-1-N-1-WT cells. Cell nuclei were stained with DAPI (blue). Abbreviations: fatty acid-binding protein 5 (FABP5), small proline-rich protein 1A (SPRR1A), small proline-rich protein 1B (SPRR1B), Involucrin (IVL), Loricrin (LOR), Filaggrin (FLG).

Microarray analysis showed that the expression of keratinocyte differentiation markers, including Involucrin (IVL), Loricrin (LOR), and Filaggrin (FLG) in A431-HBp17-KO2 cells, was 2.1 to 2.9 times higher than those of A431-WT cells. In proteomic analysis, IVL expression in A431-HBp17-KO2 cells was 1.9-fold higher than that of A431-WT cells. According to microarray and proteomic analysis, the mRNA and protein expression of FABP5 and SPRR1B clearly increased in A431-HBp17-KO2 cells compared to A431-WT cells. On the basis of microarray/GO and PPI analysis, the expression of terminal differentiation-related molecules (IVL, LOR, and FLG), and SPRR1A, which is also related to cornification and epidermal development, in addition to FABP5 and SPRR1B in A431-HBp17-KO2 and HO-1-N-1-HBp17-KO1 cells were further verified by both qRT-PCR and Western blot analysis. A qRT-PCR analysis revealed that the mRNA expression of FABP5, SPRR1A, SPRR1B, IVL, LOR, and FLG were upregulated in A431-HBp17-KO2 cells (Figure 7A). Western blot also showed that FABP5, SPRR1A, SPRR1B, and IVL expression in A431-HBp17-KO2 cells, which were upregulated in proteomic analysis, increased compared to those in A431-WT cells (Figure 7C). HO-1-N-1-HBp17-KO1 cells exhibited almost the same results as A431-HBp17-KO2 cells, but HO-1-N-1-HBp17-KO1 cells showed almost no expression of SPRR1A and SPRR1B (Figure 7B,D). Immunofluorescent staining confirmed that IVL expression was elevated in A431-HBp17-KO2 and HO-1-N-1-HBp17-KO1 cells (Figure 7E).

## 4. Discussion

An association between FGF or the FGF receptor and tumorigenicity has been reported in salivary gland cancer, hepatocyte cell carcinoma, and melanoma [24,25,26]. Fibroblast growth factor-1 and -2, which play an important role in SCC and OSCC cell growth, are secreted extracellularly, although there is no signal sequence in FGFs [27,28,29,30]. The secretory mechanism of FGFs is still unknown [31,32]. Therefore, HBp17, which was originally co-purified with FGF-2 from the A431 CM and was found to bind to FGF-1 and FGF-2 in a noncovalent and reversible manner, is considered to be a key molecule regulating the extracellular availability of FGFs [33,34,35].

Clusters of regularly interspaced short palindromic repeats (CRISPR) and CRISPR-associated protein 9 (Cas9) system, a gene-editing technology, can cut double strands of DNA and delete, replace, or insert any place in the genome sequence. It has been used for genome editing or modification not only in humans and mice but also in bacteria, parasites, and zebrafish [36,37,38]. In this study, to analyze the function of HBp17, we used the CRISPR/Cas9 technique to delete *HBp17* from the A431 and HO-1-N-1 cell lines. No morphological changes in A431-HBp17-KO and HO-1-N-1-HBp17-KO cells were observed compared to parental WT cells. The HBp17-KO cells decreased growth rates and a lower colony-forming ability compared to A431-WT and HO-1-N-1-WT cells in serum-free culture. In addition, A431-HBp17-KO1 cells produced much smaller tumors compared to A431-WT cells an in vivo assay using immunodeficient nude mice. Further, A431-HBp17-KO2 cells lost their tumorigenic potential. These results are consistent with our previous reports that decreased HBp17 expression inhibits tumor growth by inhibiting angiogenesis [16]. Liu et al. reported that *HBp17* overexpression in the A431 subclone, designated A431-#4, which has no *HBp17* expression and no tumorigenic ability in nude mice, induced tumorigenicity [12]. Tumors derived from A431-WT cells had large variability in tumor growth compared to those from A431-HBp17-KO2 cells. Gross findings showed that A431-WT cell tumors were more prone to tumor necrosis and invasive into the skin (data not shown). It is considered that the invasive manner (tumor growth externally or internally) of each tumor makes a difference in tumor size. Taken together, the results show that loss of *HBp17* expression in SCC and OSCC cells clearly inhibits tumor cell growth in vitro and in vivo.

The amount of FGFs in the CM resulted from the ability of HBp17 to release bound FGF from the ECM. By treatment with high-salt or heparinase-like enzymes, FGF-2 can be released from the ECM, including perlecan which is served as a reservoir [39]. Fibroblast growth factors released by HBp17 play an important biological role in normal and pathological tissues in an autocrine or paracrine manner [40]. Rosli et al. reported that treatment of OSCC cells with 1α,25(OH)_2_D_3_ inhibits HBp17 expression and decreases FGF-2 secretion into the CM [14]. Furthermore, higher FGF-2 levels are present in the CM of A431-#4 cells overexpressing HBp17 compared to parental cells [12]. In this study, the loss of *HBp17* resulted in a decrease in the amount of soluble FGF-2 in the CM, although intracellular FGF-2 levels did not change. This result clearly shows that HBp17 is a key molecule related to extracellular secretion of FGF-2 lacking a signal sequence, as mentioned previously [14,15].

We next focused on the altered global gene and protein expressions in A431-HBp17-KO2 cells compared to A431-WT cells. Cell differentiation molecules (FABP5, SPRR1A, AKR1C3, AKR1C2, and SPRR1B) and immune response molecules (S100A9 and S100A8) were upregulated in A431-HBp17-KO2 cells. Siegenthaler et al. reported that cellular FABP5 expression is twice as high under normal Ca^2+^ concentrations, which induce differentiation, compared to a low-Ca^2+^ medium, which inhibits differentiation [41]. A proteome profiling study on bladder SCC showed a decrease in FABP5 expression in less differentiated tumors [42]. Analysis of gene expression profiles in esophageal SCC showed that genes involved in squamous cell differentiation, including *SPRRs* and calcium-binding S100 proteins (*S100A8*, *S100A9*), are coordinately downregulated compared to their normal counterparts [43]. Phorbol ester 12-O-tetradecanoylphorbol-13 acetate (TPA), a potent tumor promotor, induced a significant increase in the formation of a cornified envelope as an indicator of terminal differentiation of epidermal keratinocytes [43,44,45]. From GO enrichment analysis of cDNA microarray data, some cascades related to epidermal development, keratinization, and cornification emerged as potential targets of HBp17. A series of epidermal structural proteins, including IVL, LOR, FLG, and the class of SPRRs, are synthesized to reinforce the cornified envelope [46]. We confirmed that these cornified envelope-related molecules were upregulated in A431-HBp17-KO2 cells compared to A431-WT cells. When we constructed PPI networks of molecules elevated by cDNA microarray and proteomic analysis, both networks were similar (Supplementary Figures S1 and S2). Although there are many reports on the induction of cancer cell differentiation and growth inhibition [47,48], there are almost no reports on the involvement of HBp17. It was reported that FLG expression increases in *HBp17*-KO mice [49]. Our results revealed that *HBp17* KO induces terminal differentiation of the squamous epithelium.

The expression of HBp17, which is highly expressed in SCC and OSCC cells, increases during the course of malignant transformation of squamous epithelial cells [10,12]. In this study, using cytological, DNA microarray, and proteomic analyses, we found for the first time that *HBp17* KO inhibits growth and motility of SCC and OSCC cells through induction of terminal differentiation. This is the first discovery of the novel role of HBp17, which inhibits differentiation of SCC and OSCC cells. In this regard, well-differentiated cancer cells, such as adenocarcinoma cells, show no HBp17 expression. At this point, the mechanism by which HBp17 inhibits cell differentiation is unclear. Further studies need to elucidate HBp17-interacting molecules involved in terminal differentiation of squamous epithelial cells. Our findings strongly suggest that HBp17 KO can be applied to differentiation inducing therapy for SCC and OSCC.

## 5. Conclusions

In this study, we have examined the functional role of HBp17 in A431 and HO-1-N-1 cells using the CRISPR/Cas9 technology. We have revealed that *HBp17* knockout inhibited cell proliferation, colony formation, and cell motility of the SCC cells, and that soluble FGF-2 levels in the medium conditioned by *HBp17*-knockout A431 and HO-1-N-1 cells were decreased compared to the parental cells. In addition, the molecules related to epidermal development, cornification, and keratinization were upregulated in the HBp17-KO cells. It was suggested that *HBp17* KO inhibits growth and motility of SCC and OSCC cells through induction of terminal differentiation. Based on these findings, the HBp17-targeting therapy in treating SCC and OSCC will be expected in the future. 

## Figures and Tables

**Table 1:** Top 10 GO terms of upregulated DEGs.

Description	Annotation ID	Genes Found	*p*-Value	Genes
Epidermis development, keratinization	GO:0008544, GO:0031424	10	9.37 × 10^−12^	SPRR3, SPRR2G, SPRR2D, SPRR2B, SPRR2A, SPRR1B, SPRR1A, KRT34, LCE3A, LCE3D
Keratinization	GO:0031424	14	5.05 × 10^−11^	SPRR3, SPRR2G, SPRR2D, SPRR2B, SPRR2A, SPRR1B, SPRR1A, KRT34, KRT6B, KRT1, IVL, LCE3A, LCE3D, KRTAP3-1
Epidermis development, cornification, keratinization	GO:0008544, GO:0070268, GO:0031424	9	3.99 × 10^−11^	SPRR3, SPRR2G, SPRR2D, SPRR2B, SPRR2A, SPRR1B, SPRR1A, KRT34, LCE3D
Epidermis development	GO:0008544	15	3.20 × 10^−11^	SPRR3, SPRR2G, SPRR2D, SPRR2B, SPRR2A, SPRR1B, SPRR1A, PTHLH, LAMA3, KRT34, KRTDAP, LCE3A, FABP5, LCE3D, CST6
Cornification	GO:0070268	14	2.45 × 10^−10^	SPRR3, SPRR2G, SPRR2D, SPRR2B, SPRR2A, SPRR1B, SPRR1A, KRT34, KRT6B, KRT1, IVL, FLG, RPTN, LCE3D
Keratinocyte differentiation, cornification	GO:0030216, GO:0070268	8	4.08 × 10^−10^	SPRR3, SPRR2G, SPRR2B, SPRR2A, SPRR1B, SPRR1A, IVL, FLG
Cornification, keratinization	GO:0070268, GO:0031424	12	7.17 × 10^−10^	SPRR3, SPRR2G, SPRR2D, SPRR2B, SPRR2A, SPRR1B, SPRR1A, KRT34, KRT6B, KRT1, IVL, LCE3D
Defense response to bacterium, defense response to fungus	GO:0042742, GO:0050832	8	6.50 × 10^−10^	GNLY, S100A12, S100A9, S100A8, MPO, HTN3, C10orf99, RNASE7
Epidermis development, keratinocyte differentiation, cornification, keratinization	GO:0008544, GO:0030216, GO:0070268, GO:0031424	6	1.27 × 10^−9^	SPRR3, SPRR2G, SPRR2B, SPRR2A, SPRR1B, SPRR1A
Keratinocyte differentiation, cornification, keratinization	GO:0030216, GO:0070268, GO:0031424	7	1.41 × 10^−9^	SPRR3, SPRR2G, SPRR2B, SPRR2A, SPRR1B, SPRR1A, IVL

DEGs, differentially expressed genes; GO, Gene Ontology.

**Table 2:** Top 10 GO terms of downregulated DEGs.

Description	Annotation ID	Genes Found	*p*-Value	Genes
Response to estradiol, response to peptide hormone, positive regulation of cell differentiation	GO:0032355, GO:0043434, GO:0045597	3	7.54 × 10^−6^	BMP7, GHR, CTGF
MAPK signaling pathway, PI3K-Akt signaling pathway, bone mineralization	hsa04010, hsa04151, GO:0030282	3	7.54 × 10^−6^	ATF4, FGFR2, FGFR3
Cell projection organization, cerebellum development	GO:0030030, GO:0021549	3	7.54 × 10^−6^	HAP1, TTBK2, C5orf42
Cell-cell signaling, positive regulation of cell population proliferation, protein phosphorylation	GO:0007267, GO:0008284, GO:0006468	3	7.54 × 10^−6^	ADAM10, FGFR2, FGFR3
Apoptotic process, endocytosis, multicellular organism development	GO:0006915, hsa04144, GO:0007275	3	7.54 × 10^−6^	FGFR2, FGFR3, DAB2
Signal transduction, cytokine–cytokine receptor interaction, tumor necrosis factor-mediated signaling pathway, Rheumatoid arthritis	GO:0007165, hsa04060, GO:0033209, hsa05323	3	2.97 × 10^−5^	TNFRSF11A, TNFSF13, LTB
Cell–cell signaling, positive regulation of ERK1 and ERK2 cascade, bone mineralization	GO:0007267, GO:0070374, GO:0030282	3	2.97 × 10^−5^	GPNMB, FGFR2, FGFR3
Pathways in cancer, multicellular organism development, bone mineralization, signaling pathways regulating pluripotency of stem cells	hsa05200, GO:0007275, GO:0030282, hsa04550	3	2.97 × 10^−5^	AXIN2, FGFR2, FGFR3
Pathogenic Escherichia coli infection, axon guidance, regulation of cell shape	hsa05130, GO:0007411, GO:0008360	3	2.97 × 10^−5^	CYFIP1, MYH10, FYN
Animal organ morphogenesis, multicellular organism development, positive regulation of transcription by RNA polymerase II, embryonic pattern specification	GO:0009887, GO:0007275, GO:0045944, GO:0009880	3	2.97 × 10^−5^	BMP7, MEIS2, FGFR2

DEGs, differentially expressed genes; GO, Gene Ontology.

## Data Availability

The data presented in this study are available from the corresponding author upon reasonable request.

## Supplementary Materials

The following are available online at https://www.mdpi.com/article/10.3390/cancers13112684/s1, Figure S1: Predicted PPI networks of upregulated DEGs in A431-HBp17-KO2 cells from microarray analysis data. Figure S2: Predicted PPI networks of upregulated DEGs in A431-HBp17-KO2 cells from proteome analysis data. Figure S3: Original Western blots and densitometric analyses relative to Figure 1. The figure shows the original Western blots shown in Figure 1, as indicated, as well as the corresponding densitometric analyses. Figure S4: Original Western blots and densitometric analyses relative to Figure 2. The figure shows the original Western blots shown in Figure 2, as indicated, as well as the corresponding densitometric analyses. Figure S5: Original Western blots and densitometric analyses relative to Figure 7. The figure shows the original Western blots shown in Figure 7, as indicated, as well as the corresponding densitometric analyses.
