# Supplementary figures and images for: Heparin-Binding Protein 17/Fibroblast Growth Factor-Binding Protein-1 Knockout Inhibits Proliferation and Induces Differentiation of Squamous Cell Carcinoma Cells
Source: Cancers (Basel). 2021 May 29;13(11):2684. doi: 10.3390/cancers13112684 (PMC8199440; doi:10.3390/cancers13112684)

Figure S1 Shintani T et. al.

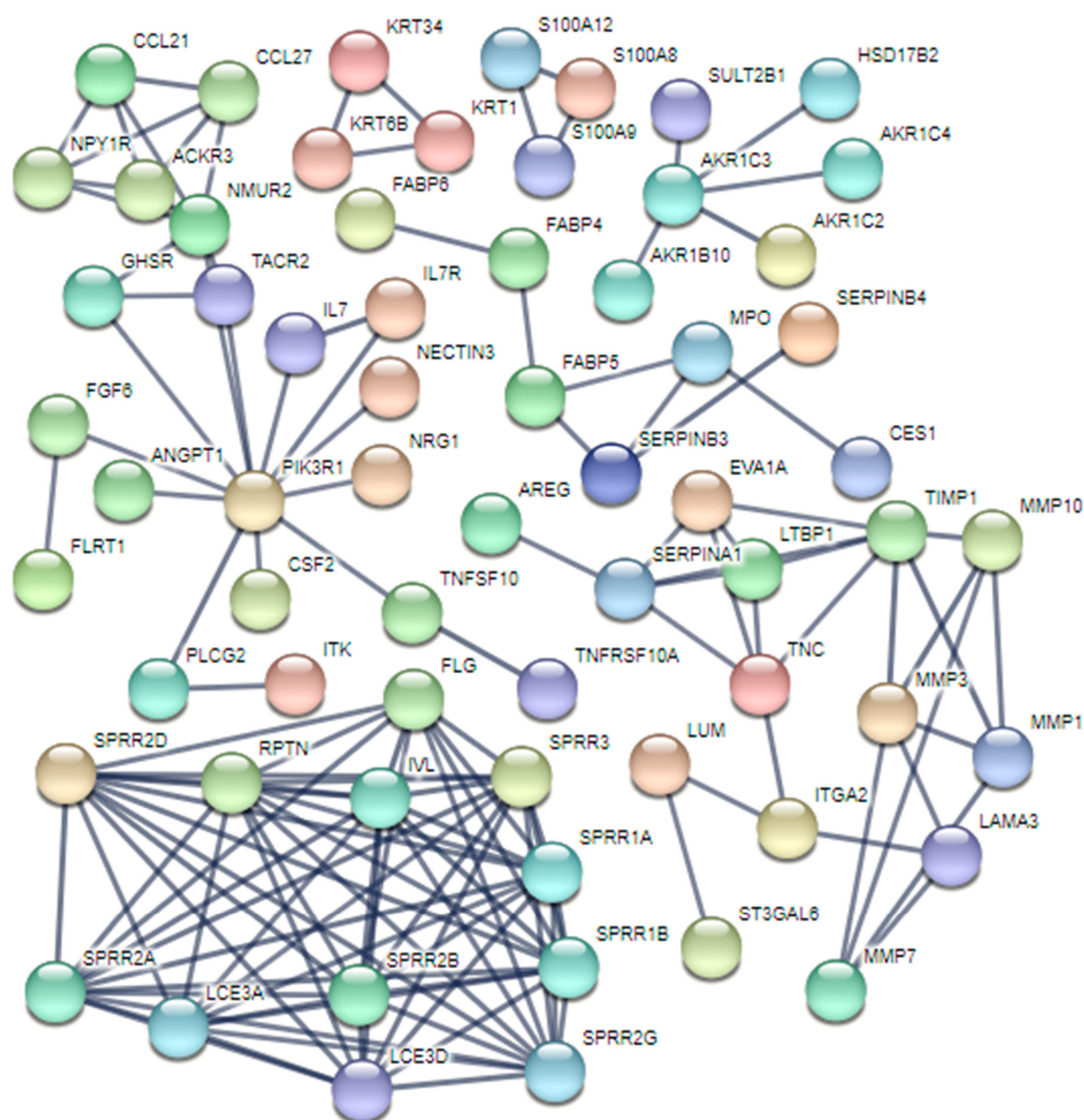



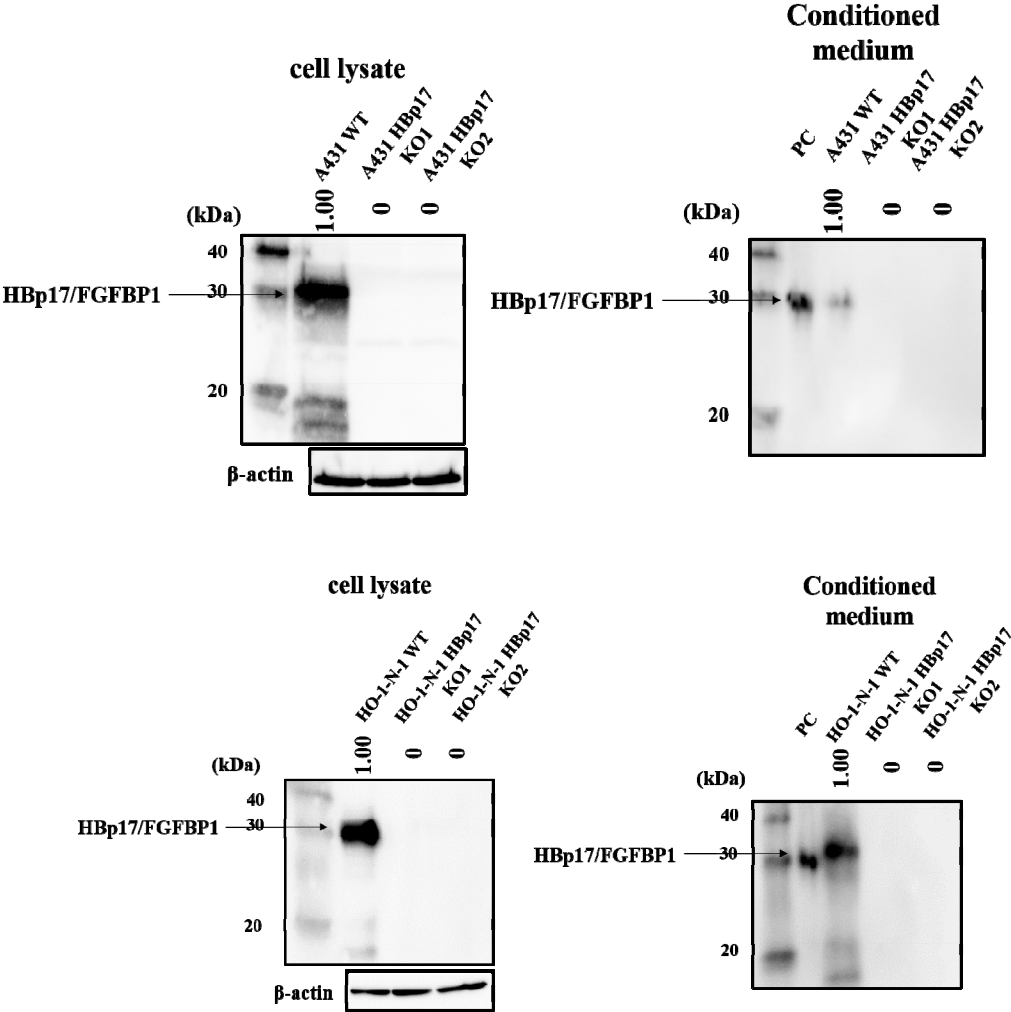

Figure S4 Shintani T et. al.

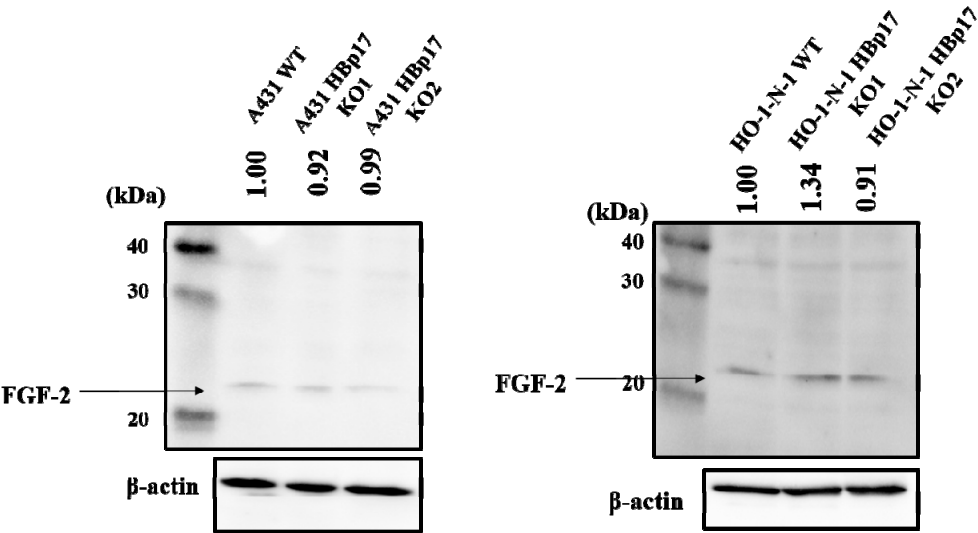

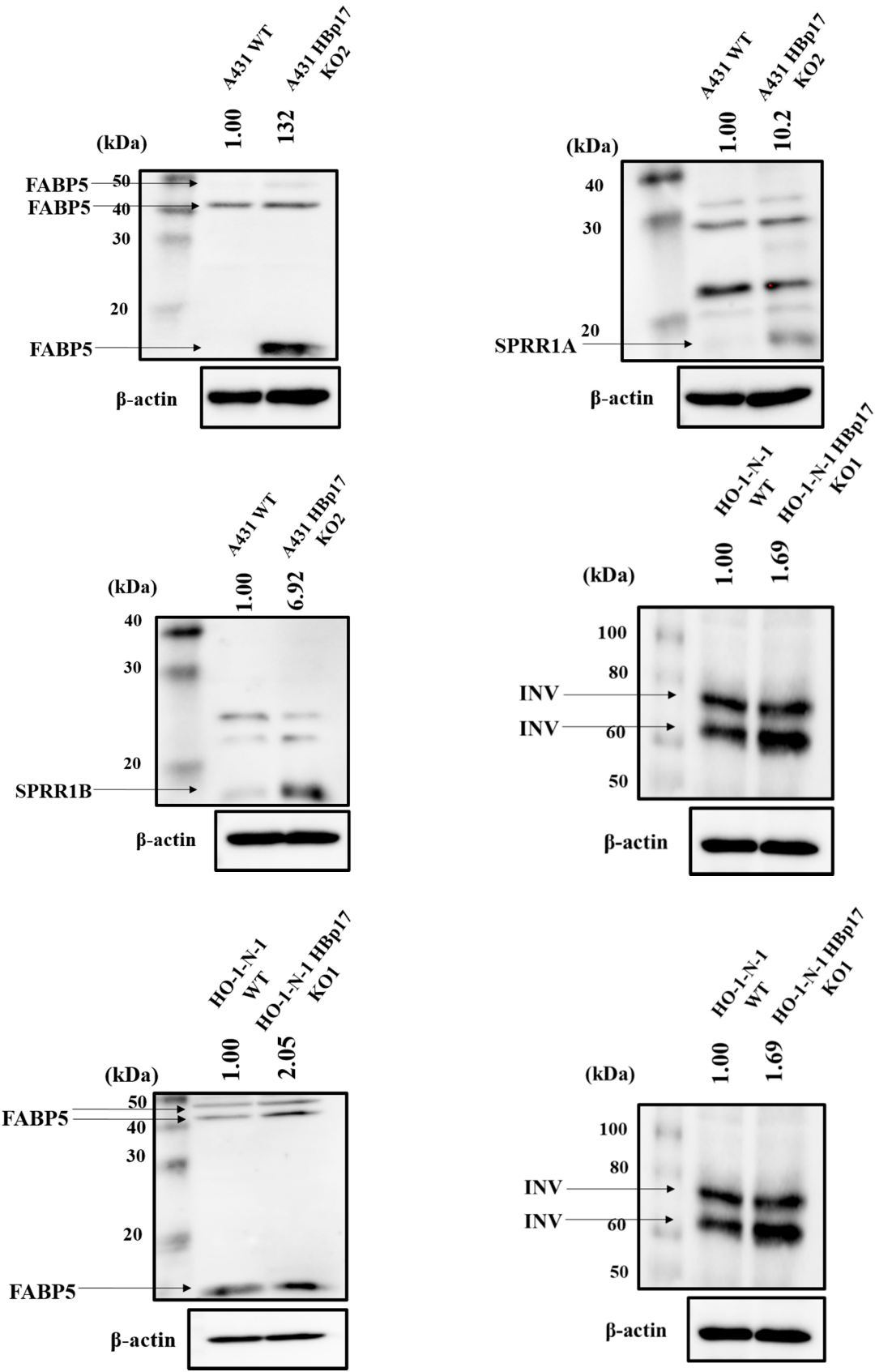

Supplement: Supplementary file 1 [file cancers-13-02684-s001.zip › cancers-1213946-supplementary.pdf]
